# Stationarity of the inter-event power-law distributions

**DOI:** 10.1371/journal.pone.0174509

**Published:** 2017-03-27

**Authors:** Yerali Gandica, João Carvalho, Fernando Sampaio dos Aidos, Renaud Lambiotte, Timoteo Carletti

**Affiliations:** 1 Department of Mathematics and Namur Center for Complex Systems—naXys, University of Namur, Namur, Belgium; 2 Centre for Physics of the University of Coimbra (CFisUC), Department of Physics, Coimbra, Portugal; University of Sussex, UNITED KINGDOM

## Abstract

A number of human activities exhibit a bursty pattern, namely periods of very high activity that are followed by rest periods. Records of these processes generate time series of events whose inter-event times follow a probability distribution that displays a fat tail. The grounds for such phenomenon are not yet clearly understood. In the present work we use the freely available Wikipedia’s editing records to unravel some features of this phenomenon. We show that even though the probability to start editing is conditioned by the circadian 24 hour cycle, the conditional probability for the time interval between successive edits at a given time of the day is independent from the latter. We confirm our findings with the activity of posting on the social network Twitter. Our results suggest that there is an intrinsic humankind scheduling pattern: after overcoming the encumbrance of starting an activity, there is a robust distribution of new related actions, which does not depend on the time of day at which the activity started.

## Introduction

The digital media are an important component of our lives. Nowadays, digital records of human activity of different sorts are systematically stored and made accessible for academic research. Hence a huge amount of data has become available throughout the past couple of decades, which allows for a quantitative study of human behaviour. For a long time, scholars from different backgrounds have been studying this field. However, some interesting and basic properties have still been outside the reach of researchers, mainly for lack of large amounts of reliable data. The increasing amount of data that is being gathered in this digital age is progressively opening up new possibilities for quantitative studies of these features. One such aspect, detected by means of data-gathering, is human bursty behaviour, which is an activity characterized by intervals of rapidly occurring events separated by long periods of inactivity [1]. The dynamics of a wide range of systems in nature displays such a behaviour [2].

Given the highly non-linear nature of human actions, their study could hence benefit from the insights provided by the field of complex systems. For the human being, the bursty behaviour phenomenon has been found to modulate several activities, such as sending letters, email messages and mobile text messages, as well as making phone calls and browsing the web [3–7]. The first works in this field suggested a decision-based queuing process, according to which the next task to be executed is chosen from a queue with a hierarchy of importance, in order to explain the observed behaviour. Different kinds of hierarchies were tested, such as the task length and deadline constraints [1, 3, 4]. Later on, Malmgren *et al*. [6, 8, 9] argued that decision making is not a necessary component of the bursty human activity patterns. Instead, they maintained that this feature is caused by cyclic constraints in life and they proposed a mechanism based on the coupling of a cascading activity to cyclic repetition in order to explain it. Nonetheless, recently, Hang-Hyun Jo *et al*. [7] applied a de-seasoning method to remove the circadian cycle and weekly patterns from the time series, and obtained similar inter-event distributions, before and after this filtering procedure. In this way, the authors concluded that cyclic activity is also not a necessary ingredient of bursty behaviour.

The goal of the present work is to contribute to the issue of human burstiness universality, by studying Wikipedia editing and Twitter posting. In particular, we show that similar inter-event distributions take place independently of the hour of the day. We relate this kind of universality, which is the result of a single person’s decision, to a kind of resource allocation (attention, time, energy), distributed in proportion to the different activities that the individual is able to do at specific times, and which is responsible for the broad distribution of inter-events, characteristic of a bursty behaviour. The bursty nature independence on the high or low activity, as a result of circadian patterns is an important issue when trying to predict human activity in social media platforms [10–12].

## Methods

Our study explores the editing activity of the super-editors (defined hereafter) in four separate Wikipedias (WP) [13], written in four different languages: English (EN-WP), Spanish (ES-WP), French (FR-WP) and Portuguese (PT-WP). In all cases, the data span a period of about ten years, ending between 2010 and 2011 (depending on the language). Each entry in the database contains the edited WP page name, the time stamp of the saving and the identification of the editor who performed the changes. Moreover, we discarded entries associated to IPs and bots, thus considering only editors who login before editing, so that the editor is univocally identified.

**Fig 1:**
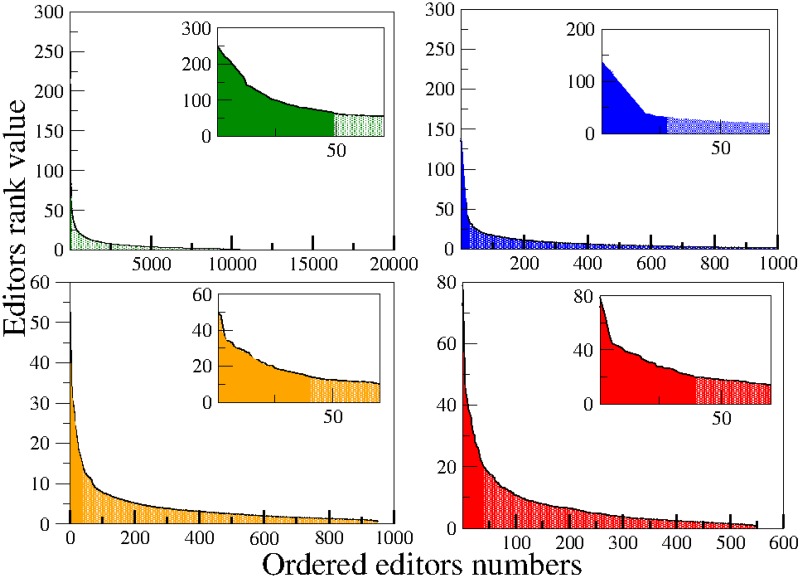
Normalized activity for all the editors with more than 2000 edits, for the four WP’s. The Wikipedia written in English is shown in the left upper panel, the one written in Spanish is in the right upper panel, in French in the left bottom panel and in Portuguese in the right bottom panel. The darker areas show the super-editors zone. The inset in each figure displays a zoom for a better visualization of the super-editors region. Some editors in the darker zone were not considered super-editors because they edited for less than one year.

Only editors with more than 2000 edits are considered. This number is large enough to reduce the impact of outliers and small enough to have a statistically relevant number of active editors in the data set. After the filters, the universe of our sample is composed by 10473 editors in EN-WP, 1110 in ES-WP, 955 in FR-WP and 551 in PT-WP. We define the normalized activity of an editor as his total number of edits divided by the total number of days since he started to edit until the last day in our data. We arrange the editors in decreasing (or, at least, non-increasing) order of their normalised activity and define each editor’s rank as his position in this ordered set (e.g. the editor with rank 1 is the editor with highest normalised activity). Super-editors, in a given language, are defined as the editors whose normalized activity is more than 25% greater than the average normalized activity in that particular WP and that have started to edit more than one year before the last day in our data. The number of super-editors is 20 in EN-WP, 10 in ES-WP, 15 in FR-WP and 24 in PT-WP. We have checked that neither WP-bots nor blocked editors are among the super-editors in our list. In Fig 1 we plot the normalized activity for all the editors with more than 2000 edits as a function of their rank for the four WP’s. The Wikipedia written in English is shown in the left upper panel, in Spanish in the right upper panel, in French in the left bottom panel and in Portuguese in the right bottom panel. The darker areas in the plots show the regions where the super-editors lie. In each figure, we include an inset that contains a zoom with a better view of the super-editors zone. Note that we have not applied the one-year of activity filter yet, so some editors in the darker zone were not considered super-editors. We focused on super-editors because their high activity provides suitable statistics; moreover, as recently shown [14], their behaviour is quite similar to the behaviour of standard editors with respect to the memory coefficient *M* and the burstiness parameter *B*, as defined in [4].

## Results

In [15] we have shown that WP editing is strongly influenced by the circadian cycle, as reported before by Yasseri *et al*. [16]. Here we analyze whether these circadian patterns have consequences on the inter-event probability distribution, namely we check whether the time between edits depends on the hour of the day at which the first edit has been carried out. To perform such an analysis we compute the conditional probability distribution for the inter-event time, considering that the first event has taken place within a specific hour of the day. If this conditional probability depends on that hour of the day, then we can conclude that circadian cycles have an influence on the human inter-event time and thus the origin of burstiness can possibly be ascribed to this dependence. In the opposite case we can conclude that burstiness in WP editing does not depend on the periodically changing conditions.

**Fig 2:**
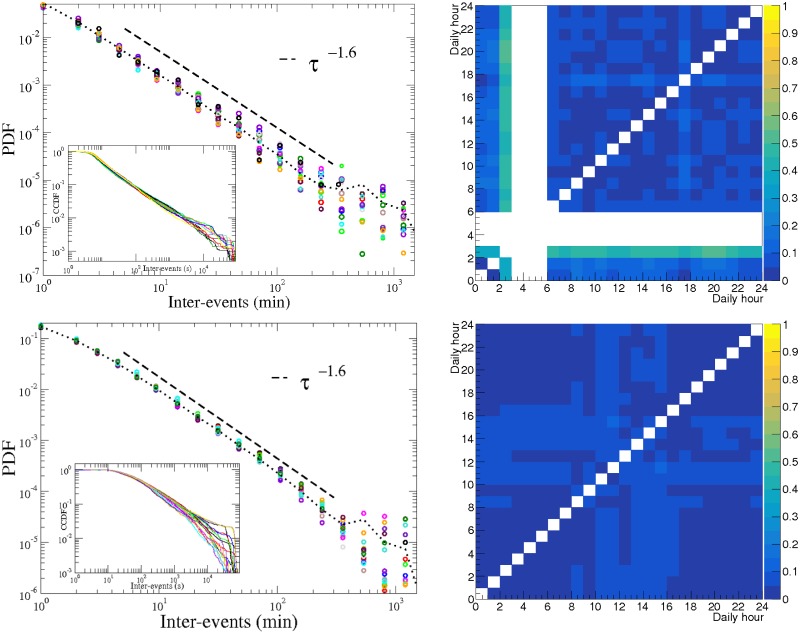
WP editing inter-event PDF and K-S distance. Left panels: PDF for the inter-event time. Right panels: K-S distance between the one-hour window CCDFs. We represent, for two of the most active WP editors (raw data in Unix time available in S1 and S2 Datasets), the probability density function to have an inter-event activity of duration *τ*, given the hour of the day at which such action is started, represented by a different color-symbol for 17 one-hour windows. The remaining windows were left out because the low statistics they contain was insufficient to draw statistically sound conclusions. The dotted lines represent the PDF using a window of 24 hours, containing all the data. One can clearly note the similar fat-tails in all the time windows, indicating they are mainly independent from the circadian cycle. In both panels, the dashed lines represent the power law best fits. In the inset is shown the CCDF for the same data. The K-S distances shown on the right panel correspond to the CCDF shown in the inset of the left panel.

Results reported in left panels of Fig 2 support the latter hypothesis. In these panels we show the probability density distribution (PDF), computed for several one-hour windows—large enough to contain adequate statistics—exhibit a similar fat tail. Note that only 17, out of 24, time windows are shown in Fig 2 left panels; 7 windows have low activity, and data are insufficient to have reliable statistical conclusions in these windows. Because the editing time stamp on the data is taken from the WP servers, and the editors can be in different time zones, we are not able to know which time windows were neglected for each user. However, we associated them with the rest periods shown in Fig 2 of [15]. The maximum inter-event duration has been fixed to 1440 min (24 hours) in order to avoid an overlap with the same hour of the following day. Our fits were done using the software ROOT [17] and compared with the procedure for fitting power-law distributions to empirical data [18] by Clauset *et al*. [19]. Anderson-Darling [20] and Kolmogorov-Smirnov [21] (K-S) tests accepted the hypothesis of Pareto distributions for the exponents showed in Fig 2 with a significance level of 5%.

In the right panels of Fig 2, we show the K-S distances (maximum distance) between each pair of complementary cumulative distribution functions (CCDF) represented in the inset of the corresponding left panel, although in this case we are showing all possible pairs of the 24 one-hour time windows. For the upper panel editor, the hours [3–6] do not have data and the hours [1–2, 7] have low statistics, therefore the [1–7] hours were the discarded hours in the corresponding left panel. For the lower panel editor the discarded hours were [10–16]. The low values of the K-S distances point out the similarity between the distributions, being the small differences related to the tails. For the second editor the tails seem to be grouped into two types of behaviour, depending on the higher or lower probability of occurrence of large inter-events, therefore naturally dividing the hours into two groups. This separation was not clearly visible in the first editor’s results.

Our results seem to indicate that, although the probability to start editing is strongly influenced by circadian rhythms, the conditional probability distribution for the time between successive edits is indeed rather independent from the time of day when the edits happen. This suggests that the bursty nature of the process is mostly independent from the circadian patterns. Note that a similar result, but on longer time scales, has been previously presented in [22], where the authors reported the robustness of the inter-event time distributions using 12 hour windows for binary contacts between conference participants.

The use of one-hour time windows is, in our opinion, a good proxy to demonstrate the stationarity of the inter-event distribution during the day. One should use even smaller time windows, but this would require a very large data sample to have enough statistics in each small period of time. The conditional probability to continue an action has been previously simulated by means of cascades of events, triggered by the initial event, which is conditioned by circadian patterns, by Malmgren *et al*. in [8].

**Fig 3:**
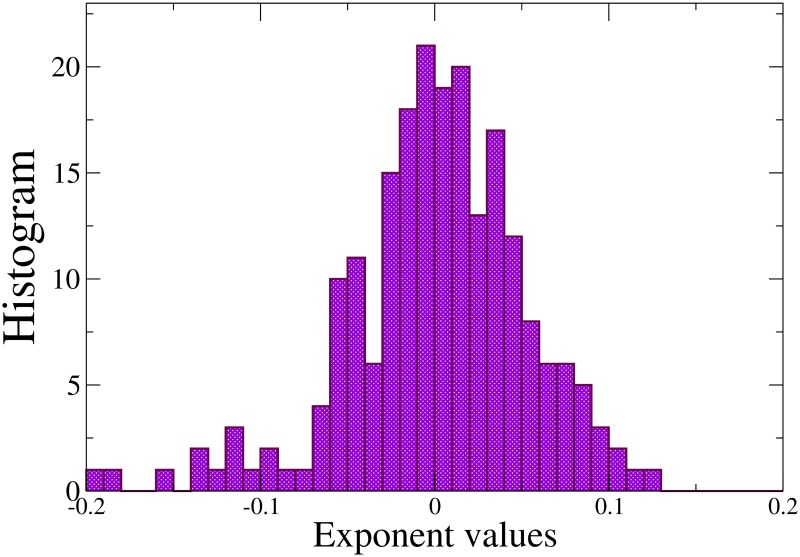
Distribution of the relative deviation on the power law exponents. $(\alpha_{i}^{\left(j\right)}-\langle \alpha^{\left(j\right)}\rangle )/\langle \alpha^{\left(j\right)}\rangle$, for all super-editors and time windows with adequate statistics, in the EN-WP.

The fat-tail distributions presented in Fig 2 can be well described by a power law *P*(*τ*|*n* ≤ *t* < *n* + 1) = *cτ*^*α*^, where *P* is the conditional probability density function for the inter-event time *τ*, on condition that the event that initiates *τ* takes place at time *t*, which lies between hour *n* and hour *n* + 1 of the day. *n* can take any integer value from 0 up to 23 and *c* and *α* are constant. The exponent *α* < 0 is independent of the hour of the day *n*. In order to study the variability of the exponent values for different super-editors and time windows, we fit, for each super-editor, *j*, and each time window, *i*, the data for the probability density function and obtain the power law exponent $\alpha_{i}^{\left(j\right)}$. We hence obtain the average exponent $\langle \alpha^{\left(j\right)}\rangle =\sum_{i}\;\alpha_{i}^{\left(j\right)}/N_{j}$, being *N*_*j*_ the number of windows used for super-editor *j*, and finally we compute the relative deviation $(\alpha_{i}^{\left(j\right)}-\langle \alpha^{\left(j\right)}\rangle )/\langle \alpha^{\left(j\right)}\rangle$, for each time window and for each super-editor. A histogram with the probability distribution of the relative deviations for the EN-WP is shown in Fig 3. The behaviour is well described, apart from some fluctuations, by a normal distribution with standard deviation 0.055.

**Fig 4:**
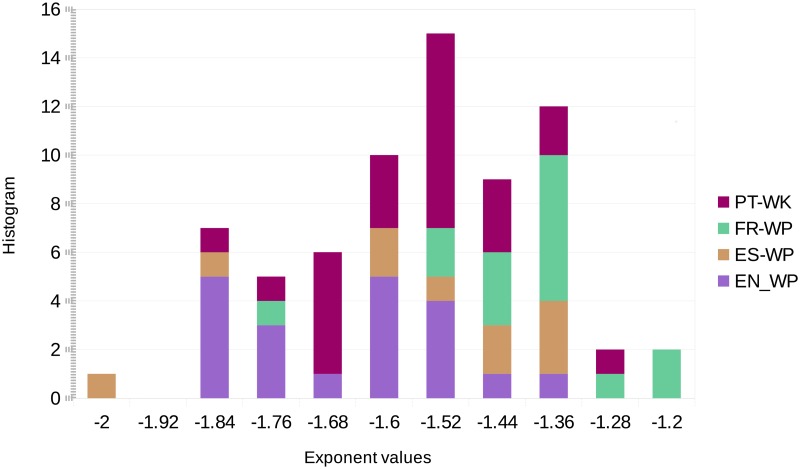
Distribution of the average exponent values 〈*α*^(*j*)^〉 for the super-users in the four WP’s.

We report the distribution of the average exponents for all inter-events, 〈*α*^(*j*)^〉, for super-editors in the four WP’s, in Fig 4. We notice that the average value is −1.59; note that in [23] the average value of the exponents computed using twenty-four-hour long windows for the 100 most active WP editors, was reported to be −1.44.

In trying to understand the origin of the robustness of the power-law distribution among the different hours of the day, one may be tempted to affirm that it is caused by the saving mechanism; the editor would be constantly saving his work as a precaution against power or network failure, something that could be of behavioral origin across human activity. This hypothesis can explain the independence of the inter-event time distribution with respect to the starting time. However, short inter-event times were found regularly between edits of different pages by the same editor, which means that the short inter-event times are not just a consequence of the simple act of saving the work done so far in a certain WP page. Still, the saving mechanism cannot be entirely discarded as the source of burstiness as there is some probability, albeit small, that the editor opens several windows and edits several pages in parallel.

### Stationarity of the inter-event power-law distributions in other data

**Fig 5:**
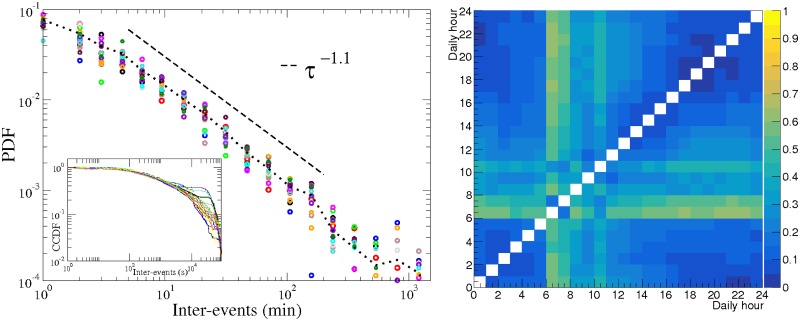
Tweets inter-event PDF and K-S distance. A)PDF for the inter-event duration of all the tweets posted by one user (raw data in Unix time available in S3 Dataset). B) K-S distance between the one-hour window distributions. Each color-symbol in the left panel represents the probability distribution of all the inter-events registered in each one-hour window. Seven windows were left out because the low statistics they contain was insufficient. The dotted lines represent the inter-event probability using a window of 24 hours, containing all the data. The dashed line represents the power law best fit. The inset shows the CCDF for the same data. The K-S distances shown on the right panel correspond to the CCDF shown in the inset of the left panel.

We have tested these findings in a different human activity, in order to check that it is not an effect exclusive to WP editing. We chose the activity of posting in the social micro-blogging platform, Twitter. This online social networking service enables users to send and read short 140-character messages called “tweets”. This on-line platform is very recent and good statistics for one-hour window time series is rarely freely available. One such rare case is shown in Fig 5, for which the possibility of being a robot or an organization was discarded.

In the left panel of Fig 5 (Fig 5A), we show the inter-event PDF, computed in different one-hour windows, for all the tweets posted by one representative user, starting in February 2010. Following the same procedure as before, we discard the hourly intervals of low activity (hours [6–13]), and we use a twenty-four-hour cut-off as well. In the right panel of the same figure (Fig 5B) we show the K-S distance between all pairs of one-hour window CCDFs, showed in the inset of the left panel.

In this case, the K-S distances are higher than those found for the Wikipedia editors, as a consequence of the lower statistics. However, a similarity was found for most of the comparisons of pairs of the non-discarded one-hour time windows. Also in this data, Anderson-Darling [20] and Kolmogorov-Smirnov [21] tests accepted the hypothesis of Pareto distributions for the power-law exponent 1.1 with a significance level of 5%.

## Discussion

To summarize, in this work we provide numerical evidence that the conditional probability, *P*(*τ*|*n* ≤ *t* < *n* + 1), to have an inter-event of duration *τ* after an edit of WP registered at time *t*, such that *n* ≤ *t* < *n* + 1 is mainly independent from *n*. Moreover, this probability is fat-tailed and well described by a power law. It could be related to some sort of queuing process, but we prefer to see it as due to a resource allocation (attention, time, energy) process, which exhibits a broad distribution: shorter activities are more likely to be executed next than the longer ones, which ultimately may be responsible for the bursty nature of human behaviour.

Using the data for the editing of WP and for the activity of tweeting, our results seem to indicate that there is an intrinsic mechanism to human nature: before performing an action (make a phone call, send a tweet, edit Wikipedia, etc) we must overcome a “barrier”, acting as a cost, which depends, among many other things, on the time of day. However, once that “barrier” has been crossed, there exists a robust distribution of activities, which no longer depends on the time of day. Our findings suggest that the bursty nature of human beings is mainly independent of circadian patterns, in agreement with the results found, using a different method, by Hang-Hyun *et al*. [7]. This result could open the perspective to applications less specific than the study of Wikipedia or Twitter. Future work includes simulations taking into account circadian patterns to reproduce the probability to perform an action, while maintaining a constant conditional probability distribution for the time between successive events.

## Supporting information

S1 Dataset Data, in Unix time, for user on top panel of Fig 2. (TXT)

S2 Dataset Data, in Unix time, for user on bottom panel of Fig 2. (TXT)

S3 Dataset Data, in Unix time, for user on Fig 5. (TXT)
